# The Male Staff of Hospitals

**Published:** 1912-01-20

**Authors:** 


THE MALE STAFF OF HOSPITALS.

The Secretary of the Bradford Royal Infirmary, and the Secretary of the Chesterfield Hospital and Dispensary, are thanked for their communications on this subject. Will hospital secretaries in general kindly supply us with similar information, which must in its completed form materially help each hospital when they have to determine the best course to take in regard to the National Insurance Act?

